# *In silico* Biological Activity of Steroids from the Marine Gastropods *Telescopium telescopium* Collected from South West Coast of India

**Published:** 2018

**Authors:** A.S. Ragi, P.P. Leena, K.J.P. Prashob, S.M. Nair

**Affiliations:** 1. Department of Chemical Oceanography, Faculty of Marine Sciences, Cochin University of Science and Technology, Cochin-682016, India; 2. Inter University Center for Development of Marine Biotechnology, Cochin University of Science and Technology, Cochin-682016, India

**Keywords:** CLC-Pred, PAAS, Sterols, *Telescopium telescopium*

## Abstract

**Background:**

The purpose of this study was to investigate the sterol profiling and predict the pharmacological potential of marine gastropod *Telescopium telescopium (T. telescopium)*, collected from mangrove ecosystem in the South west coast of India.

**Methods:**

Sterol fractions were separated from the crude lipids using 15% ethyl acetate. Ethyl acetate fractions were dried under ultrahigh purity N_2_ and analyzed using GC-MS. The biological activity was predicted using the software CLC-Pred; *In silico* predictions of cytotoxicity for tumor and non-tumor cell lines and PASS.

**Results:**

This study proved the existence of four sterols, of which cholesterol was abundant. It was found that most of the steroids profiled from *T. telescopium* displayed activity against reproductive system as well as skin related diseases.

**Conclusion:**

The predicted anti infertility and skin related activity of the steroids identified from the marine gastropod *T. telescopium* is useful to attract industrial interest towards this species which will be helpful in rising new combinations with added therapeutic and nutritional worth.

## Introduction

Molluscs are the second largest phylum in the marine environment and are divided into seven classes. Gastropods, Bivalves, Cephalopods and Polyplacophora are the major four classes, which include snails, clams, scallops, oysters, cuttlefish, and octopus 1. Marine gastropods are unique, as they are the distinctive class of organisms that have long been recognised as a vital source capable of biosynthesising wide range of secondary metabolites, including Alkaloids, Peptides, Triterpenes and Steroids 2–4.

Gastropods and bivalves are also regarded as excellent sources of nutritionally important compounds such as Fatty acids, Amino acids and Sterols 2,5–7. Among the various compounds isolated, sterols from the marine gastropods are remarkably unique, which exhibit wide range of structural variation in steroidal structure. Sterols are a highly diverse group of metabolically active compounds and their presence in dietary compounds advocate important strategy in prevention and treatment of cancer 1,8,9. In the gastropods, usually conventional steroids are derived through three different ways: (1) *de novo* biosynthesis of sterols without the incorporation of mevolonate and acetate, (2) filtration activity with or without successive changes by gastropods and (3) symbiosis with microorganism present in the surrounding environment 5,10. Sterols are naturally occurring compounds usually having a 1, 2-cyclopenta-no-phenthren skeleton with a 3-hydroxyl group 11, which occur in marine organisms as complex inseparable mixtures. Researchers have keen interest in these marine sterols since 1960, due to their importance in pharmacological industry. Phytosterols prevent the production of carcinogens, cancer-cell growth, invasion and metastasis, and promote apoptosis of cancerous cells 12. Positive impact of sterols on human health is mainly due to their hypocholesterolemic activity, which arises from their marked similarity with cholesterol. Previous reports suggest that phytosterols have anti-cancerous effects against skin, stomach, lung, ovary and estrogen-dependent human breast cancer 12–14.

The main goal of this work was extending the knowledge on sterols from marine gastropods especially from the genus *Telescopium*. There are no investigations on the steroid composition of *Telescopium telescopium (T. telescopium)* collected from Pappinisseri mangrove estuary. Novel biological potential of these steroids has been projected with the assistance of computer aided drug discovery methodology. The software PASS (Prediction of Activity Spectra for Substances) 15,16 has been used to identify the possibility of the steroids identified in the present study, in clinical application.

## Materials and Methods

A total of two hundred specimens of mangrove snails, *T. telescopium* were collected from Pappinisseri mangrove ecosystem, (Latitude: 11° 56′ 8″ E and Longitude: 75° 21′ 13″ N) situated in the Kannur district that was covering a distance of 7–8 *km* from the coastline. *T. telescopium* was collected by hand picking and washed in tap water and kept in deep freezer until further analysis in the laboratory. The washed specimens were rinsed with distilled water and the soft tissues were removed from the shells. Freeze dried tissue samples were crushed by using a mortar and pestle and were shaken vigorously to produce homogeneity.

For the analysis of steroids, finely powdered freeze dried tissue samples were extracted for 48 *hr* with a mixture of cold Chloroform-Methanol (2:1, *v/v*) using temperature controlled shaker 17. For the complete extraction, the above procedure was repeated twice or thrice and extracts were combined and evaporated to dryness using rotary evaporation. The extracted residue was subjected to mild alkaline hydrolysis using 0.5 *M* KOH/MeOH and gentle heating (70°*C* for 6 *hr*). After cooling, the neutral lipids were partitioned from the alkaline solution into 2 *ml* of Hexane, which was separated and stored for further analysis.

Sterol fraction eluted with 15% ethyl acetate in 100 *ml* n-hexane using silica gel column was evaporated to 1 *ml* under ultra high purity N_2_. Analysis was carried out using a Perkin Elmer Clarus GC 620 GC, equipped with MS detector and a non-polar HP ultra-double-fused silica capillary column (30 *m*, 0.32 *mm* internal diameter, 0.25 *μm* film thickness). Operating conditions were as follows: ion source of electron voltage 70eV kept at 200°*C* spectra were scanned from 50 to 600 *m/z* with a scan time of 1.50 *s*. Initially, the temperature was increased from 50 to 220°*C* at a rate of 10°*C* per *min* and held at 220°*C* for 5 *min*. Then the temperature was again increased from 220 to 290°*C* at a rate of 1°*C* per *min* and held at 290°*C* for 10 *min*. The detector was held at 290°*C* and helium was used as carrier gas. Full data was obtained with the use of MS Turbo Mass version 5.3.2. Individual compounds were identified by comparison of mass spectra with literature and library data, retention time of authentic standards and interpretation of mass spectrometric fragmentation patterns.

The pharmacological potential of the steroids was identified and *in silico* predictions were made using the software PASS (Prediction of Activity Spectra of Substances) 16,18,19.

## Results

**Figure 1.**
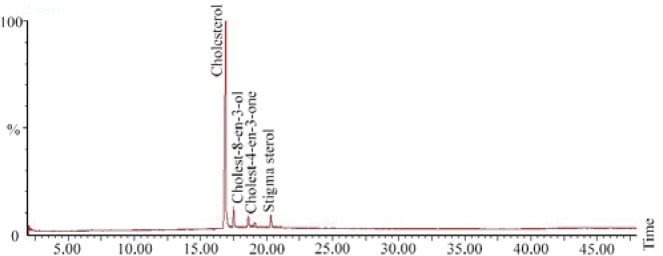
Total ion chromatogram of sterols from *T. telescopium.*

**Figure 2.**
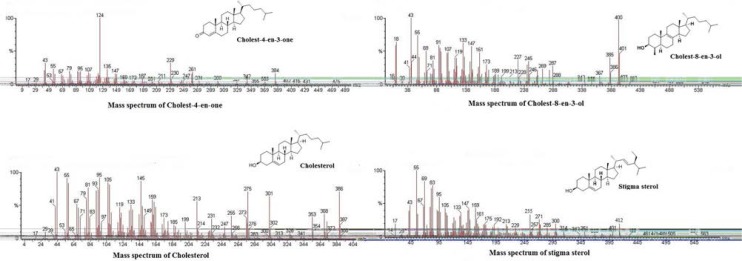
Structure and mass spectrum of steroids.

Sterols present in the soft tissue of mangrove gastropod *T. telescopium* were separated from crude lipid fraction using column chromatography followed by acetylation using pyridine and BSTFA. The derivatized fractions were analyzed using GC MS which yielded three types of sterols and one sterone. Sterols of *T. telescopium* are generally complex mixtures that are composed of C27, C28 and C29 sterols. All the three compounds were already reported, of which cholesterol (79%) was the major sterol and cholest-8-en-3-ol 13, cholest-4-en-3-one (2%) and stigma sterol (6%) were identified. The results of percentage abundance were represented in table 1. Chromatogram of sterols was represented in figure 1. Peak 1 is cholesterol, identified by using Electron Impact Mass Spectrometry (EIMS) which showed *m/z* with (relative intensity) values: 386 (72), 368(36), 353(28), 301(65), 275(68), 255(30), 231 (25), 213(35), 55(86), 43(100). Peak 2 corresponds to cholest 8-en-3-ol and its ElMS *m/z* (relative intensity) values were 385(38), 367(16), 287(8), 245(12), 227 (14), 161(18), 91(28), 81(46), 69(52), 55(64), 43(100). Peak 3, ElMS *m/z* (relative intensity) values were 384 (30), 369(5), 342(10), 299(8), 261(35), 229(42), 187 (25), 124(100) corresponding to cholest-4-en-3-one. Peak 4 is stigma sterol and corresponding ElMS *m/z* (relative intensity) values were: 412(20), 364(3), 255 (16), 213(9), 199(8), 159(25), 145(29), 133(26), 105 (32) 91(34), 83(64), 69(52), 55(100), 41(39). Structure and mass spectrum of the identified steroids were represented in figure 2.

**Table 1.** Percentage abundance of steroids from *T. telescopium*

**Compound**	**% abundance of steroids from *T. telescopium***
**Cholesterol**	79.49±0.84
**Cholest-8-en-3-ol**	12.62±0.67
**Cholest-4-en-3-one**	1.93±0.51
**Stigmasterol**	5.96±0.51

**Figure 3.**
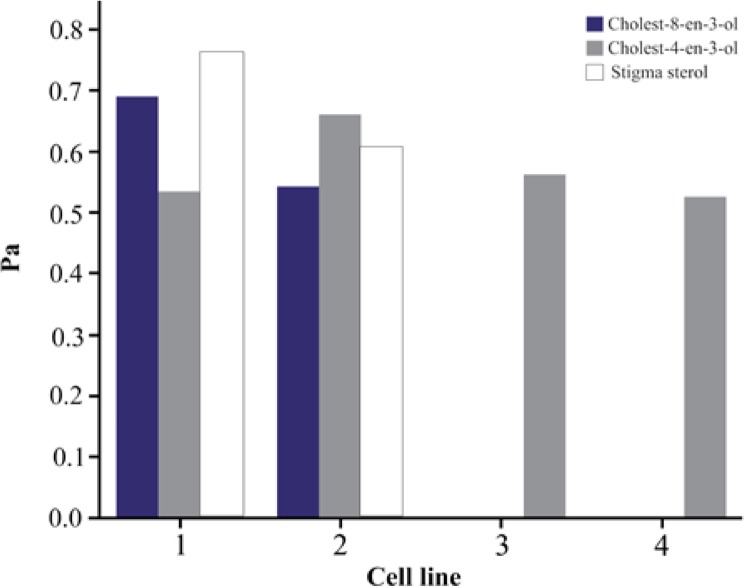
Cancer cell line prediction of steroids from *T. telescopium.* 1. Stomach adeno carcinoma cells, 2. Gastric carcinoma cells, 3. Lung carcinoma cells, 4. Gastric epithelial carcinoma cells.

*In silico* predictions were made using the software PASS (Prediction of Activity Spectra of Substances). The predicted values were presented through its probability to be active (Pa) or inactive (Pi) data. Among the steroids obtained, cholest-8-en-3-ol and cholest-4-en-3-one showed activity against stomach adenocarcinoma and gastric carcinoma cells (Figure 3). Stigma sterols showed activity against stomach adenocarcinoma cells, gastric carcinoma cells, lung carcinoma cells and gastric epithelial carcinoma cells and their respective Pa values were presented in figure 3. Steroids in this study were mainly regarded as hypolipidemic, respiratory analeptic, anti-infertility in females, antieczematic, antipruritic, dermatologic and prostate disorders treatment agents (Table 2). Cholest-8-en-3-ol is found to exhibit all the above said activities. Stigma sterol did not exhibit a wider range of activities, but it showed the uncommon activities such as anti-psoriatic and cancer chemo preventive activities. The p-values ranging from 0.8 to 0.9 are expected to have high activity and values from 0.7 to 0.79 are considered as unlikely to cover some positive biological activity. The p-values <0.7 reflect certain biological activity.

**Table 2.** *In silico* activity of Sterols from Marine gastropod, *T. telescopium*

**Sl. No.**	**Activity**	**Cholest-8-en-3-ol**	**Cholest-4-en-3-one**	**Stigma sterol**
**1**	Adenomatous polyposis treatment	++	+	+
**2**	Hypolipemic	++	−	+++
**3**	Respiratory analeptic	++	++	−
**4**	Anti-infertility, female	++	+	−
**5**	Antieczematic	++	+	++
**6**	Antipruritic	++	++	+
**7**	Dermatologic	+	+	++
**8**	Bone diseases treatment	+	+	+
**9**	Antiosteoporotic	+	+	+
**10**	Analeptic	+	+	−
**11**	Immunosuppressant	+	+	+
**12**	Prostate disorders treatment	+	+	+
**13**	Proliferative diseases treatment	−	+	+
**14**	Chemopreventive	−	−	++
**15**	Antipsoriatic	−	−	+

+++ Indicate Pa values >0.9; ++ Indicate Pa values ranging from 0.8 to 0.9; + Indicate Pa values ranging from 0.7 to 0.79; −< Indicate Pa values 0.7.

## Discussion

This is the first time reporting the four steroids viz, cholesterol, cholest-8-en-3-ol, cholest-4-en-3-one and stigma sterol from the gastropod, *T. telescopium.* This study is in good agreement with the previous reports on sterols in adult cephalopods and gastropods 20–22. Cholesterol was the major sterol in the soft tissue of *T. curta*, marine gastropod from Neendakara estuary 23. Generally, several gastropod species have been shown to possess the ability for de novo synthesis of cholesterol. Previous reports on gastropods show that they are capable of dealkylating some phytosterols to cholesterol 5. It is possible that most gastropods do not require a dietary source of cholesterol for growth 5. Earlier reports on gastropods collected from Illawarra Coast, Australia show maximum abundance for cholesterol and other sterols were found in very low concentrations 22. As stated by Bergmann W *et al* and Benkendorff K *et al* 20,22, cholesterol is the only sterol found in cephalopods. Numerous other sterols were detected in the gastropod spawn but in such small quantities that most remain unidentified.

For the analysis of pharmacological potential of the steroids identified in the present study, *in silico* predictions were made using the software PASS (Prediction of Activity Spectra of Substances) 10,15,17,18. The recent version of PASS predicts all together 3,678 kinds of activity with around 95% of mean accuracy and prediction 24. The expected values for biological activities were acquired by comparing the natural structures of each compound with structures or sub structures of more than 30,000 well known biologically active drugs. The predicted values were presented through its probability to be active (Pa) or inactive (Pi) data 10.

Among the steroids obtained, cholest-8-en-3-ol and cholest-4-en-3-one showed activity against stomach adenocarcinoma and gastric carcinoma cells. Stigma sterols showed activity against stomach adenocarcinoma cells, gastric carcinoma cells, lung carcinoma cells and gastric epithelial carcinoma cells. Gastric cancer is the third most common cause of cancer-related death in the world, and it remains difficult to be cured in Western countries 25. Stigma sterol isolated from *Azadirachta indica (A. indica*) showed chemo preventive activity against skin cancer 26. In recent years, high level of cholesterol in blood causes severe problems such as heart diseases, heart attack and stroke in human beings. Both stigma sterol and cholest-8-en-3-ol show hypolipemic activity (lipid lowering), which reduce blood cholesterol. Cholest-8-en-3-ol shows activity against infertility in females and prostrate disorder treatments. Cholest-4-en-3-one is found to be used as central nervous system stimulants that include a wide variety of medications used to treat depression.

## Conclusion

*In silico* activity studies of identified sterols from gastropod, *T. telescopium* was investigated. It is the primary screening of pharmacological potential of sterols. Sterols especially stigma sterols and cholest-8-en-3-ol have worldwide importance in the field of cardio vascular diseases and their use in the food supplements have been approved by U.S Food and Drug Administration. The bioactivities of these sterols are well known and hence the presence of stigma sterols in *T. telescopium* promotes this organism in human diet. As no work has been done so far on *T. telescopium* from mangrove estuary of Kerala, this work is useful to attract industrial interest towards this species which will be helpful in rising new combinations with added therapeutic and nutritional worth. The predicted anti infertility and skin related activity of the steroids identified from the marine gastropod *T. telescopium*, underlines the need for extended investigations in this direction to unravel the regenerative activities of these steroids.
